# Uncertainties in pentose-phosphate pathway flux assessment underestimate its contribution to neuronal glucose consumption: relevance for neurodegeneration and aging

**DOI:** 10.3389/fnagi.2015.00089

**Published:** 2015-05-19

**Authors:** Anne-Karine Bouzier-Sore, Juan P. Bolaños

**Affiliations:** ^1^Centre RMSBBordeaux Cedex, France; ^2^Institute of Functional Biology and Genomics, University of Salamanca-CSICSalamanca, Spain

**Keywords:** astrocyte–neuron interactions, pentose-phosphate pathway, glycolysis, aging neuroscience, ^13^C-NMR spectroscopy

## The impact of PPP in redox and energy conservation

**Figure 1:**
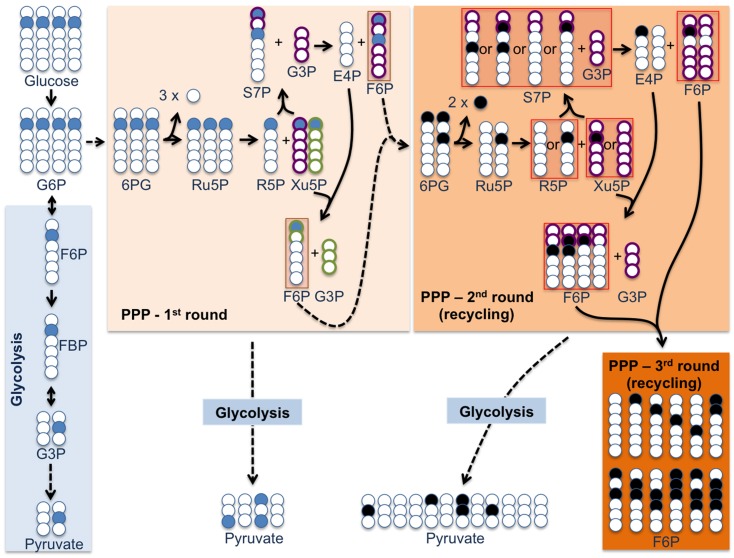
**^13^C-Pyruvate labeling pattern from [2-^13^C]glucose by *recycling* PPP underestimates PPP contribution to glucose metabolism**. Labeling pattern in ^13^C-pyruvate originated from glycolysis ([2-^13^C]pyruvate) differs from that originated from a first round of PPP ([3-^13^C]- and [1,3-^13^C_2_]pyruvate), thus helping to estimate the contribution of both pathways in glucose utilization (blue carbons). However, some cell types, such as neurons, express high PGI activity responsible for high equilibration rate of F6P with G6P, hence leading to a *recycling*-like PPP (PPP – second and third rounds). Identification of these recycling-PPP ^13^C-labeled intermediates (black carbons) identifies a more complex pattern of ^13^C-pyruvate labeling ([1-^13^C]-, [2-^13^C]-, and [1,2-^13^C_2_]pyruvate after the second PPP round; pattern after a third PPP round is omitted for simplicity) that overestimates glycolysis, hence underestimating the contribution of PPP to glucose utilization. Please note that [1-^13^C]pyruvate could also come from TCA cycle (pyruvate *re-recycling*; see text). However, this formation represents as low as one-twelfth of the pyruvate originated from the second round of PPP, thus likely minimally affecting to PPP activity estimation. Therefore, labeling in ^13^C-Ru5P and ^13^C-R5P ([2-^13^C]-, [1,2-^13^C_2_]-, [1,2,3-^13^C_3_]-, etc.) after the second PPP round can be used for an accurate estimation of PPP in highly recycling cells.

The pentose-phosphate pathway (PPP) promotes the oxidative decarboxylation of glucose-6-phosphate (G6P) in two consecutive steps, catalyzed by glucose-6-phosphate dehydrogenase (G6PD) and 6-phosphogluconate dehydrogenase (6PGD), yielding ribulose-5-phosphate (Ru5P) (Figure 1) (Wamelink et al., 2008). These steps constitute the so-called oxidative PPP branch, where the redox energy of G6P is conserved as NADPH(H^+^). Together with other NADPH(H^+^) regenerating systems, such as NADP-dependent isocitrate dehydrogenase and malic enzyme (ME), the oxidative PPP branch represents the most important source of reducing equivalents for (i) antioxidant enzymes, such as glutathione peroxidases and thioredoxin reductases, and (ii) fatty acid synthase (Dringen et al., 2007). Ru5P is isomerized into ribose-5-phosphate (R5P), which serves either as the precursor for nucleotide biosynthesis, or it continues metabolism through the non-oxidative PPP branch. In the latter, R5P epimerize into xylulose-5-phosphate (Xu5P), with which it transketolases producing sedoheptulose-7-phosphate (S7P) plus glyceraldehyde-3-phosphate (G3P). In turn, S7P and G3P transaldolase to form fructose-6-phosphate (F6P) and erythrose-4-phosphate (E4P). E4P is then transketolated with Xu5P forming F6P and G3P. Thus, through the PPP, three moles of G6P yield three CO_2_, two F6P, and one G3P. Since PPP-derived F6P and G3P are glycolytic intermediates too, they can follow conversion into pyruvate. Thus, glycolysis and PPP are two different pathways that share common pools of F6P and G3P intermediates. Accordingly, G6P converted into pyruvate through the PPP conserves both the redox and the energetic values of glucose, highlighting a yet unrecognized high impact of PPP flux activity in redox/energy conservation.

## Radioactive PPP assessment Ex vivo

Several approaches have been used to estimate the fraction of G6P that is metabolized through the PPP. For experiments performed *ex vivo* (cultured and freshly isolated cells, or tissue slices), radiometric ^14^CO_2_ detection after incubation of the biological samples in the presence of [1-^14^C]glucose, under initial velocity conditions, reports flux through the oxidative branch of the PPP. However, since C_1_-G6P is also decarboxylated in the tricarboxylic acid (TCA) cycle, the ^14^CO_2_-value, taken alone, intrinsically over-estimates PPP activity. To overcome this drawback, a parallel incubation under identical conditions needs to be performed to quantify ^14^CO_2_ collected from [6-^14^C]glucose, since C_6_-G6P is exclusively decarboxylated in the TCA after G6P is converted into pyruvate both through glycolysis and PPP. Thus, the difference in the rate of ^14^CO_2_ produced from [1-^14^C]glucose and that from [6-^14^C]glucose represents the flux of ^14^CO_2_ exclusively produced at the oxidative PPP branch (Hothersall et al., 1979; Larrabee, 1989). Whilst this approach is suitable for *ex vivo* analyses, it is technically tedious for *in vivo* PPP assessments, as CO_2_ collection *in vivo* is hardly quantitative.

## ^13^C-NMR PPP assessment *in vivo*

Several approaches have been used to assess the proportion of G6P that is metabolized through the PPP *in vivo*—which are also useful for *ex vivo*. A widely used method is essentially based on the analysis of ^13^C abundance of certain carbon-atoms in lactate upon perfusion (*in vivo*) or incubation (*ex vivo*) with [2-^13^C]glucose. Thus, the abundance of [3-^13^C]lactate is assumed to be exclusively originated *via* PPP metabolism, whereas the abundance of [2-^13^C]lactate, *via* glycolysis (Figure 1) (Brekke et al., 2012). In a slightly different approach based on the use of [1,2-^13^C_2_]glucose, the abundances of [3-^13^C]lactate and [2,3-^13^C]lactate are considered to be originated *via* PPP and glycolysis, respectively (Jalloh et al., 2015). [1,6-^13^C_2_,6,6-^2^H_2_]glucose has also been used to measure cerebral PPP activity *in vivo* (Ben-Yoseph et al., 1995) on the bases that it produces [3-^13^C]lactate and [3-^13^C,3,3-^2^H_2_]lactate through glycolysis, but [3-^13^C,3,3-^2^H_2_]lactate and unlabeled lactate through PPP (Ross et al., 1994). The ratios of these lactate isotopomers can be quantified using gas chromatography/mass spectrometry for calculation of PPP activity, and expressed as the percentage of glucose metabolized to lactate that passed through the PPP (Ross et al., 1994; Ben-Yoseph et al., 1995). These analyses provide information on the relative contribution of a metabolic pathway within overall glucose utilization, in contrast to the initial velocity analyses, which informs on absolute metabolic flux values. Whilst such a difference might account for the controversies in PPP activity values reported by different laboratories (Herrero-Mendez et al., 2009; Brekke et al., 2012; Rodriguez-Rodriguez et al., 2013), in our opinion there are some methodological concerns, as explained below.

## Occurrence of recycling PPP

A critical point that is often overlooked is the occurrence of G6P recycling from PPP-derived F6P. Thus, phosphoglucose isomerase (PGI) is a near-equilibrium enzyme that has been shown to be highly active at converting F6P into G6P in certain cells and/or tissues, such as, e.g., neurons (Gaitonde et al., 1989), which can re-enter the PPP (Figure 1). In such *recycling*-like PPP, only the PPP-derived G3P fully escapes from this cycle to be transformed into pyruvate trough glycolysis. High recovery of G6P from PPP-derived F6P may represent a bioenergetics advantage, since the redox energy of glucose can be conserved as NADPH(H^+^) at the expense of only one carbon (C_1_) per G6P. It should be noted that, in certain cells such as neurons, 6-phosphofructo-1-kinase (PFK1, which converts F6P into fructose-1,6-bisphosphate or F16BP) represents a glycolysis bottleneck (Almeida et al., 2004). Thus, PFK1 *in situ* activity is very low in neurons when compared, e.g., with neighbor astrocytes (Almeida et al., 2004). Such a low PFK1 activity is due to the virtual absence of 6-phosphofructo-2-kinase/fructose-2,6-bisphosphatase-3 (PFKFB3) (Herrero-Mendez et al., 2009), responsible for the formation of fructose-2,6-bisphosphate (F26BP)—the most potent positive PFK1 effector. In contrast, astrocytes abundantly express PFKFB3 protein—and F26BP concentration—and, accordingly, a high *in situ* PFK1 activity (Almeida et al., 2004). It is therefore likely that, whereas PPP-derived F6P is preferentially recycled in the PPP in neurons, it is preferably converted into lactate in astrocytes. In fact, the *in situ* PFK1 activity is ~four-fold lower in neurons when compared with astrocytes (Almeida et al., 2004). Therefore, we hypothesize that, in neurons, (i) most PPP-derived F6P is recycled back into the PPP, and (ii) the vast majority of G3P pool is originated directly from PPP activity. However, in astrocytes, G3P pool is originated from (i) G6P through glycolysis, (ii) PPP-derived F6P then *via* glycolysis, and (iii) directly from PPP activity. On our opinion, these considerations may have implications when interpreting the ^13^C-labeling data for the estimation of PPP activity, as indicated below.

## PPP assessment is intrinsically underestimated

When [2-^13^C]glucose infusion and/or incubation is used, [1-^13^C]- and [1,3-^13^C_2_]lactate abundances are exclusively PPP-derived, whereas [2-^13^C]lactate abundance is glycolysis-derived (Figure 1). Therefore, the relative abundance of [1-^13^C]- and [1,3-^13^C_2_]lactate when compared with total lactate is often considered to be the proportion of glucose metabolized through the PPP (Brekke et al., 2012). However, [1,3-^13^C_2_]F6P, formed in the PPP from [2-^13^C]glucose, when converted into [1,3-^13^C_2_]G6P *via* PGI activity (F6P → G6P-forming), which is very high in neurons (Gaitonde et al., 1989), can produce [2-^13^C]F6P in the second PPP round (Figure 1). Thus, as from the second PPP round and thereafter, such [2-^13^C]F6P will also yield [2-^13^C]lactate, largely over-estimating glycolysis. In addition, the degree of *recycling* PPP activity is not addressed when measuring [3-^13^C,3,3-^2^H_2_]lactate plus unlabeled lactate that is formed from [1,6-^13^C_2_,6,6-^2^H_2_]glucose (Ben-Yoseph et al., 1995), since this approach does not address the proportion of, and rate at which F6P is recycled back into the PPP. It may therefore be concluded that the methodological approaches to estimate recycling PPP activity may be intrinsically underestimated in neurons. We hypothesize that the degree of recycling PPP activity would be best indicated by the levels of PPP-specific intermediates (Figure 1). In view that [1,2-^13^C_2_]Ru5P is exclusively formed by recycling PPP from [2-^13^C]glucose when entering the third round, and [1,2,3-^13^C_3_]Ru5P when entering the fourth round, we propose to determine the relative abundance of mono-labeled vs. multiple labeled ^13^C-R5P + ^13^C-Ru5P, which is feasible by liquid chromatography/mass spectroscopy (LC/MS).

## Overestimation of glycolysis

It is a widely held custom to determine total lactate released from cells as an index of glycolysis. However, given that—at least under several circumstances—the precise proportion of G6P that is metabolized through PPP to lactate is uncertain, total lactate as an index of glycolysis should be taken with caution. We rather propose that lactate release should be considered as an overall index for glucose utilization—i.e., glycolysis plus PPP. To estimate glycolysis, the use of [5-^3^H]glucose conversion into ^3^H_2_O, which takes place at enolase, is often used to assess the rate of glycolysis (Neely et al., 1972). However, since enolase cannot distinguish the origin—glycolysis or PPP—of its substrate—2-phosphoglycerate—, the production of ^3^H_2_O from [5-^3^H]glucose does not help to clarify the specific contribution of glycolysis to glucose utilization. Thus, the use of [5-^3^H]glucose could greatly overestimate the rate of glycolysis in neurons, as it has been shown in the rat heart (Goodwin et al., 2001), which in turn underestimates PPP. We encourage the use of [3-^3^H]glucose to estimate glycolysis, since ^3^H of C_3_-glucose interchanges with water at aldolase (Katz et al., 1965), hence reducing the contribution of PPP to the collected ^3^H_2_O. Since PFK1 *in situ* activity is very low in neurons (Herrero-Mendez et al., 2009), the [3-^3^H]glucose approach reflects more accurately glycolysis than the [5-^3^H]glucose one. Using [3-^3^H]glucose, we have reported that primary cortical neurons produce ^3^H_2_O at a rate that is ~four-fold lower than in primary cortical astrocytes (Almeida et al., 2004). However, the proportion of glucose that is metabolized through glycolysis in neurons can be overestimated. It has been widely reported that [1-^13^C]glucose perfusion in rodents renders, in the brain, a higher ^13^C-specific enrichment in [4-^13^C]glutamate (mainly present in neurons) compared to [4-^13^C]glutamine (mainly present in astrocytes) (Fitzpatrick et al., 1990; Kanamatsu and Tsukada, 1994; Bouzier et al., 1999). When comparing the specific enrichment of [4-^13^C]glutamate and [1-^13^C]glucose, it was calculated that ~90% of the [4-^13^C]glutamate was originated from the glycolytic metabolism of [1-^13^C]glucose. The ~10% loss in specific enrichment of [4-^13^C]glutamate is usually attributed to a loss of ^13^C_1_ in the PPP. Unfortunately, this interpretation does not take into account that [3-^13^C]lactate can be actively used by neurons and converted into [4-^13^C]glutamate *in vivo* (Bouzier et al., 2000), since glycolytically-generated lactate is shuttled from astrocytes to neurons (Pellerin and Magistretti, 1994; Bouzier-Sore et al., 2006), therefore underestimating *in vivo* neuronal PPP.

## Pyruvate re-cycling

Pyruvate can be re-generated from the TCA cycle intermediates malate and oxaloacetate through ME and PEPCK, respectively. Such a pyruvate *re-cycling* has been shown to occur in neurons (Cerdan et al., 1990; Cruz et al., 1998), where malic enzyme is expressed profusely (Vogel et al., 1998; McKenna et al., 2000), although its occurrence in astrocytes has also been proposed (Olstad et al., 2007). Therefore, when using [2-^13^C]glucose to estimate the PPP activity, [2-^13^C]pyruvate entering the TCA cycle will yield [1-^13^C]pyruvate from *re-cycling*, which will be undistinguishable from that returning from the second PPP round (Figure 1). This further reinforces our proposal that PPP should be estimated by measuring ^13^C incorporation into the five carbon-atom sugars, instead of that into lactate or pyruvate.

## PPP is an advantage for neurons that may failure in neurodegeneration and aging

Neurons are deficient in antioxidant glutathione (Bolaños et al., 1995), hence recycling of reduced glutathione (GSH) from its oxidized form (GSSG) is critical for their survival. Since NADPH(H^+^) is essential for this process, the re-cycling version of the PPP—which recovers a considerable proportion of G6P—explains both the low glucose consumption and the efficient GSH regenerating activity of neurons. In contrast, astrocytes express higher PPP-rate limiting step G6PD, and PPP activity, than neurons (Garcia-Nogales et al., 2003; Herrero-Mendez et al., 2009), besides higher glycolysis (Almeida et al., 2004; Herrero-Mendez et al., 2009). This is likely indicating that both non-recycling PPP plus glycolysis contribute to glucose consumption in astrocytes. Accordingly, G6P recovery through re-cycling PPP in neurons represents an advantage for neuronal survival. A large body of evidence is now showing, by PET studies, decreased glucose consumption—actually reflecting glucose uptake—in the human brain during aging and neurodegeneration (Chen and Zhong, 2013). This observation has been widely interpreted as a putative cause of limited energy production in neurons. However, before we uncover the actual contribution of PPP to glucose consumption by the brain cells, particularly neurons and astrocytes, such an assertion remains uncertain. We herein opine that the decrease in glucose utilization occurring in aging and neurodegeneration, far from merely causing a bioenergetics problem, would be responsible for an oxidative damage due to failed PPP-derived NADPH(H^+^) regeneration in neurons. Furthermore, we also propose that the impaired glucose utilization observed in PET studies in neurodegeneration and aging reflects, mainly, reduced glucose consumption in astrocytes. This will, in turn, result in reduced lactate released for neuronal conversion into either energy or neurotransmitter glutamate. Either hypothesis, i.e., low neuronal PPP activity causing failed antioxidant capacity, low lactate supply by astrocytes, or a combination of both, may have been largely overlooked as the cause of both bioenergetics and antioxidant neuronal damage during aging and neurodegeneration. More in-depth studies to overcome the methodological drawbacks herein highlighted should be undertaken in order to unveil the actual contribution of PPP to neuronal survival *in vivo*.

### Conflict of interest statement

The authors declare that the research was conducted in the absence of any commercial or financial relationships that could be construed as a potential conflict of interest.

## Abbreviations

E4P: erythrose-4-phosphate
F6P: fructose-6-phosphate
F6B: fructose-1,6-bisphosphate
G3P: glyceraldehyde-3-phosphate
G6P: glucose-6-phosphate
R5P: ribose-5-phosphate
G6PD: glucose-6-phosphate dehydrogenase
GSH: glutathione, reduced form
GSSG: glutathione, oxidized form
F26BP: fructose-2,6-bisphosphate
ME: malic enzyme
PEPCK: phosphoenolpyruvate carboxykinase
PFK1: 6-phosphofructo-1-kinase
PFKFB3: 6-phosphofructo-2-kinase/fructose-2,6-bisphosphatase, isoform 3
PGI: phosphoglucose isomerase
6PGD: 6-phosphogluconate dehydrogenase
PPP: pentose-phosphate pathway
Ru5P: ribulose-5-phosphate
TCA: tricarboxylic acid
S7P: sedoheptulose-7-phosphate
Xu5P: xilulose-5-phosphate.
